# Astragalus Polysaccharide Alleviates Ulcerative Colitis by Regulating the Balance of mTh17/mTreg Cells through TIGIT/CD155 Signaling

**DOI:** 10.3390/molecules29010241

**Published:** 2024-01-02

**Authors:** Qi Wan, Jiaqi Huang, Qiuping Xiao, Zeyun Zhang, Zheyan Zhang, Li Huang, Yifei Deng, Bailing Deng, Haimei Zhao, Youbao Zhong, Duanyong Liu

**Affiliations:** 1 Department of Postgraduate, Jiangxi University of Chinese Medicine, Nanchang 330004, China; wanqi@jxutcm.edu.cn (Q.W.); 202081504006@jxutcm.edu.cn (J.H.); zhangzeyun@jxutcm.edu.cn (Z.Z.); zhangzheyan@jxutcm.edu.cn (Z.Z.); huangli@jxutcm.edu.cn (L.H.); 2 College of Pharmacy, Jiangxi University of Chinese Medicine, Nanchang 330004, China; xiaoqiuping@jxutcm.edu.cn; 3 College of Traditional Chinese Medicine, Jiangxi University of Chinese Medicine, Nanchang 330004, China; 202001005110@jxutcm.edu.cn (Y.D.); 20211050@jxutcm.edu.cn (B.D.); 20050852@jxutcm.edu.cn (H.Z.); 4 Laboratory Animal Research Center for Science and Technology, Jiangxi University of Chinese Medicine, Nanchang 330004, China; 5 Formula-Pattern Research Center, Jiangxi University of Chinese Medicine, Nanchang 330004, China

**Keywords:** astragalus polysaccharide, colitis, mTh17/mTreg cells, TIGIT/CD155 signaling pathway

## Abstract

The balance between memory Th17 cells (mTh17) and memory Treg cells (mTreg) plays a key role in the pathogenesis of ulcerative colitis (UC), and TIGIT signaling is involved in the differentiation of mTh17/mTreg cells. Astragalus polysaccharide (APS) has good immunomodulatory and anti-inflammatory effects. Here, the regulatory effects and potential mechanisms of APS on mTh17/mTreg cells in UC are explored. A UC model was induced with dextran sulfate sodium (DSS) and treated simultaneously with APS (200 mg/kg/day) for 10 days. After APS treatment, the mice showed a significant increase in colonic length and a significant decrease in colonic weight, colonic weight index and colonic weight/colonic length, and more intact mucosa and lighter inflammatory cell infiltration. Notably, APS significantly down-regulated the percentages of Th17 (CD4^+^CCR6^+^), cmTh17 (CD4^+^CCR7^+^CCR6^+^) and emTh17 (CD4^+^CCR7^−^CCR6^+^) cells and significantly up-regulated the percentages of cmTreg (CD4^+^CCR7^+^Foxp3^+^) and emTreg (CD4^+^CCR7^−^Foxp3^+^) cells in the mesenteric lymph nodes of the colitis mice. Importantly, APS reversed the expression changes in the TIGIT molecule on mTh17/mTreg cells in the colitis mice with fewer CD4^+^CCR6^+^TIGIT^+^, CD4^+^CCR7^−^CCR6^+^TIGIT^+^ and CD4^+^CCR7^−^CCR6^+^TIGIT^+^ cells and more CD4^+^Foxp3^+^TIGIT^+^, CD4^+^CCR7^−^Foxp3^+^TIGIT^+^ and CD4^+^CCR7^−^Foxp3^+^TIGIT^+^ cells. Meanwhile, APS significantly inhibited the protein expression of the TIGIT ligands CD155, CD113 and CD112 and downstream proteins PI3K and AKT in the colon tissues of the colitis mice. In conclusion, APS effectively alleviated DSS-induced UC in mice by regulating the balance between mTh17/mTreg cells, which was mainly achieved through regulation of the TIGIT/CD155 signaling pathway.

## 1. Introduction

Th17/Treg cell imbalance is the classic paradigm of ulcerative colitis (UC) pathogenesis, and reshaping this balance is an effective measure for treating UC [1,2]. Clinically, UC is characterized by symptoms such as diarrhea, abdominal pain and hematochezia, which tend to be recurrent and chronic in nature [3,4]. Immune memory plays a role in maintaining long-term homeostasis in the gut and preventing inflammatory responses induced by repeated exposure to homologous antigens [5]. Importantly, the balance between memory Th17 (mTh17) and memory Treg (mTreg) cells plays an important role in the onset, remission and relapse of UC [6,7]. These memory cells are characterized by their rapid and robust response compared to that of naïve cells with mTh17 cells facilitating swift eradication of infections and mTreg cells enhancing immune tolerance to avert inflammatory reactions in the gut [8]. CCR7^+^ memory T cell surface receptors contribute to cell entry into sites of inflammation, express lymph node homing receptors, effectively stimulate dendritic cells, assist T cells and differentiate into CCR7^−^ memory T cells upon re-exposure to homologous antigens [9,10]. According to the expression of CCR7, mTh17 cells can be divided into CCR7^+^ central-memory Th17 (cmTh17) and CCR7^−^ effector-memory Th17 (emTh17) cells. Similarly, mTreg cells can be divided into cmTreg and emTreg cells. Increasingly, studies have shown that the balance between mTh17/mTreg cells is disrupted in colitis [11,12] with higher levels of mTh17 cells and lower levels of mTreg cells. Therefore, this suggests that regulating the balance between mTh17 and mTreg cells is a pivotal therapeutic strategy for the management of UC.

TIGIT, an emerging immune checkpoint, is expressed on natural killer (NK) cells and various T cell subsets, including memory and activated T cells, CD4^+^T cells, CD8^+^T cells, Treg cells and follicular T helper cells (Tfh) [13,14]. As a co-inhibitory molecule, TIGIT plays an important role in the function, proliferation and differentiation of T cells [15], including mTh17 and mTreg cells. TIGIT has the ability to enhance immunosuppression in Treg cells, and elevated levels of TIGIT^+^Treg cells have been known to suppress Th1 and Th17 cell responses [16,17]. TIGIT specifically binds to multiple ligands, including CD155, CD112 and CD113. Extensively, CD155, CD112 and CD113 are over-expressed in several solid tumors (pancreatic cancer, glioblastoma, colon cancer, ovarian cancer, etc.) and affect cell proliferation, migration invasion and adhesion by regulating tumor-associated signaling pathways [18,19,20,21]. Blocking TIGIT/CD155 signaling can reverse T cell depletion and enhance T cell activity and effector function to effectively exert anti-tumor abilities. Critically, CD155 binds TIGIT with the highest affinity and promotes direct and indirect down-regulation of the T cell response [20]. CD113 can suppress the activity of T cells [22], effectively blocking CD112R and its ligand CD112 signaling pathway, thus promoting the activation and proliferation of T cells and NK cells [23]. Nevertheless, the role of TIGIT/CD155 signaling in the balance of mTh17/mTreg cells is not yet known and neither is its pathogenetic role in UC.

Astragalus membranaceus, also known as yellow ginseng, blood ginseng, etc., is a Chinese medicine that tonifies the middle, benefits the qi, stops sweating, induces diuresis and swelling, removes toxins, generates muscle and is widely used in clinical practice [24]. Polysaccharide is an important product of natural medicinal plants, and its preparation in a reasonable way can better utilize the medicinal efficacy of the raw material. Astragalus polysaccharide (APS) is one of the main active ingredients of Astragalus, which has various biological activities, such as antiviral, immunomodulatory and antioxidant, in addition to low toxicity, low resistance, low residue and no contamination [25]. Currently, the few studies on APS intervention in UC are mainly focused on the inhibition of NLRP3 inflammatory vesicles and the NF-κB signaling pathway and enhancement of Treg cell responses [26,27]. Our research group had initially discovered that APS could effectively promote an increase in the proportion of Treg cells within Peyer’s patches in the intestinal tract of rats with TNBS-induced colitis [28]. In another study that utilized a DSS-induced mouse model of colitis, we found that APS could regulate the expression of inflammatory cytokines, including IL-2, IL-6, IL-12p70, IL-23 and TNF-α, in the colonic tissues of mice with colitis. APS also appeared to reshape the homeostasis of Tfh/Treg cells. UC clinical pathogenesis is characterized by prolonged and recurrent episodes, and mTh17/mTreg cells play a key role in maintaining UC remission and relapse. However, it did not significantly affect the regulation of Th17 cells [29]. Herein, we further elucidate the action mechanism of APS through TIGIT/CD155 signaling to regulate the balance of mTh17/mTreg cells for the treatment of UC.

## 2. Results

### 2.1. APS Alleviates DSS-Induced UC in Mice

**Figure 1:**
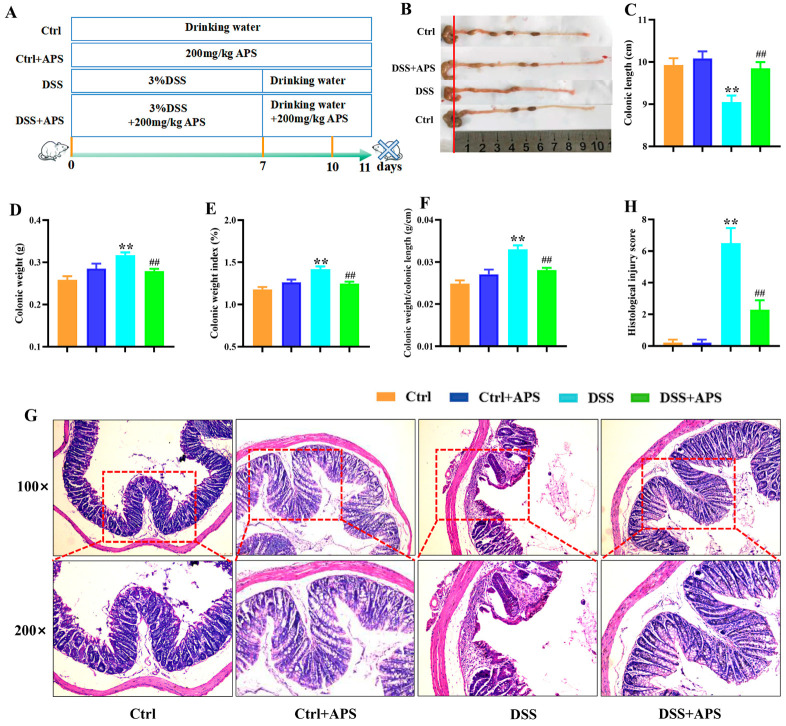
Therapeutic evaluation of Astragalus polysaccharide on DSS-induced experimental colitis mice. (**A**) Replication of the experimental colitis mice model and administration of APS. (**B**) Representative images of the mice colons observed with the naked eye. (**C**) Colonic length. (**D**) Colonic weight. (**E**) Colonic weight index. (**F**) Colonic weight/colonic length. (**G**) Histological morphology of the colonic tissue (H&E staining, magnification 50× or 100×, Bar = 250 μm or 75 μm). (**H**) Histological scores of these four groups of mice. Data are expressed as mean ± SEM (*n* = 10). Significantly different compared to the Ctrl group (** *p* < 0.01). Significantly different compared to the DSS group (^##^ *p* < 0.01).

UC is characterized by clinical symptoms such as weight loss, blood in the stool and diarrhea, and the pathology is characterized by ulcer formation and inflammatory cell infiltration [30]. Here, the colitis mice showed a distinct colonic inflammatory response with shortening, colon weight gain (Figure 1B–E) and apparent pathological damage of ulcer formation and inflammatory cell infiltration (Figure 1G). Compared with the DSS group, the DSS+APS group showed a significant increase in colonic length (Figure 1B,C) and a significant decrease in colonic weight (Figure 1D), colonic weight index (Figure 1E) and colonic length/colonic weight (Figure 1F); under the light microscope, it was observed that the colitis mice treated with APS had more intact colonic mucosa, fewer inflammatory cells and intact goblet cells, and their pathological injury scores were also significantly decreased (Figure 1G,H). These results indicated that APS was effective in alleviating DSS-induced experimental colitis.

### 2.2. APS Regulates the Balance between mTh17 and mTreg Cells in Colitis Mice

**Figure 2:**
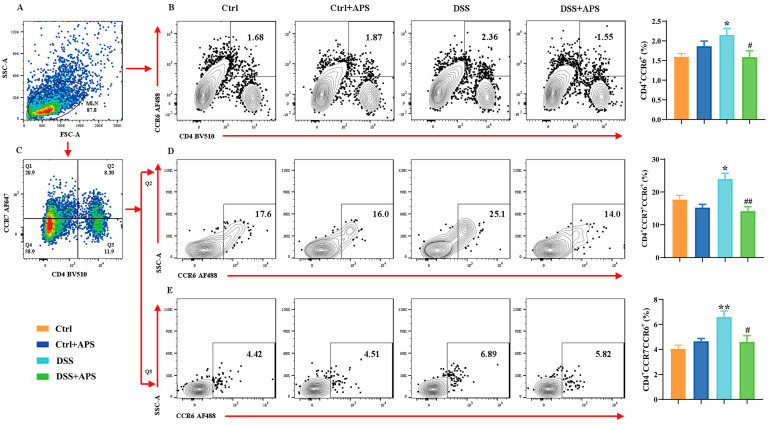
Flow cytometry analysis of mTh17 cells. (**A**) Total number of lymphocytes in representative samples. (**B**) CD4^+^CCR6^+^ cells (Th17) in representative samples. (**C**) CD4^+^CCR7^+^/CD4^+^CCR7^−^ cells in representative samples. (**D**) CD4^+^CCR7^+^CCR6^+^ cells (cmTh17) in representative samples. (**E**) CD4^+^CCR7^−^CCR6^+^ cells (emTh17) in representative samples. Data are expressed as mean ± SEM (*n* = 10). Significantly different compared to the Ctrl group (* *p* < 0.05, ** *p* < 0.01). Significantly different compared to the DSS group (^#^ *p* < 0.05, ^##^ *p* < 0.01).

**Figure 3:**
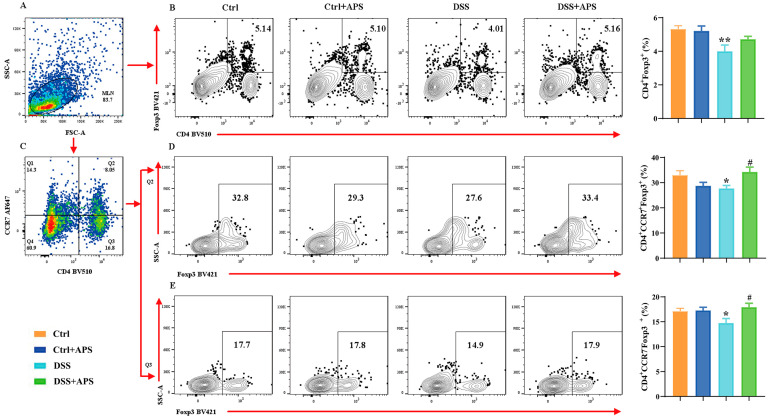
Flow cytometry analysis of mTreg cells. (**A**) Total number of lymphocytes in representative samples. (**B**) CD4^+^Foxp3^+^ cells (Treg) in representative samples. (**C**) CD4^+^CCR7^+^/CD4^+^CCR7^−^ cells in representative samples. (**D**) CD4^+^CCR7^+^Foxp3^+^ cells (cmTreg) in representative samples. (**E**) CD4^+^CCR7^−^Foxp3^+^ cells (emTreg) in representative samples. Data are expressed as mean ± SEM (*n* = 10). Significantly different compared to the Ctrl group (* *p* < 0.05, ** *p* < 0.01). Significantly different compared to the DSS group (^#^ *p* < 0.05).

It is well known that the Th17/Treg cell balance plays a critical role in the development of UC. Here, the DSS-induced colitis mice showed a remarkable imbalance with a significant increase in CD4^+^CCR6^+^ Th17 cells (Figure 2B) and a significant decrease in CD4^+^Foxp3^+^ Treg (Figure 3B) cells in the mesenteric lymph nodes (MLNs). Critically, the colitis mice also exhibited an abnormal imbalance of mTh17/mTreg cells with higher frequencies of CD4^+^CCR7^+^CCR6^+^ cmTh17 (Figure 2D) and CD4^+^CCR7^−^CCR6^+^ emTh17 cells (Figure 2E) and lower frequencies of CD4^+^CCR7^+^Foxp3^+^ cmTreg (Figure 3D) and CD4^+^CCR7^−^Foxp3^+^ emTreg cells (Figure 3E) in the MLNs. Meanwhile, the percentages of CD4^+^CCR7^+^CCR6^+^ cmTh17 (Figure 2D) and CD4^+^CCR7^−^CCR6^+^ emTh17 (Figure 2E) cells were significantly lower in the DSS+APS group than in the DSS group, while CD4^+^CCR7^+^Foxp3^+^ cmTreg (Figure 3D) and CD4^+^CCR7^−^Foxp3^+^ emTreg cells (Figure 3E) were significantly higher. These results indicated that APS effectively regulated the balance of mTh17/mTreg cells in the colitis mice.

### 2.3. APS Can Effectively Regulate TIGIT Expression on mTh17/mTreg Cells

**Figure 4:**
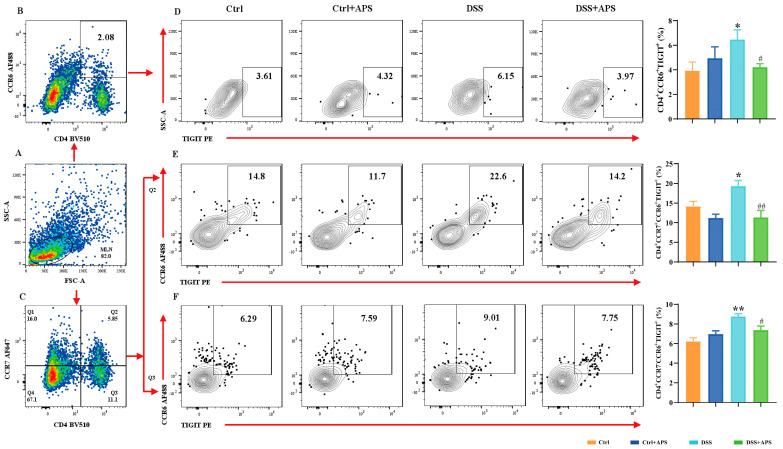
Flow cytometry analysis of the expression of TIGIT on the surface of mTh17 cells. (**A**) Total number of lymphocytes in representative samples. (**B**) CD4^+^CCR6^+^ cells (Th17) in representative samples. (**D**) CD4^+^CCR6^+^TIGIT^+^ cells (TIGIT^+^ mTh17) in representative samples. (**C**) CD4^+^CCR7^+^/CD4^+^CCR7^−^ cells in representative samples. (**E**) CD4^+^CCR7^+^CCR6^+^TIGIT^+^ cells (TIGIT^+^cm-mTh17) in representative samples. (**F**) CD4^+^CCR7^−^CCR6^+^TIGIT^+^ cells (TIGIT^+^em-mTh17) in representative samples. Data are expressed as mean ± SEM (*n* = 10). Significantly different compared to the Ctrl group (* *p* < 0.05, ** *p* < 0.01). Significantly different compared to the DSS group (^#^ *p* < 0.05, ^##^ *p* < 0.01).

**Figure 5:**
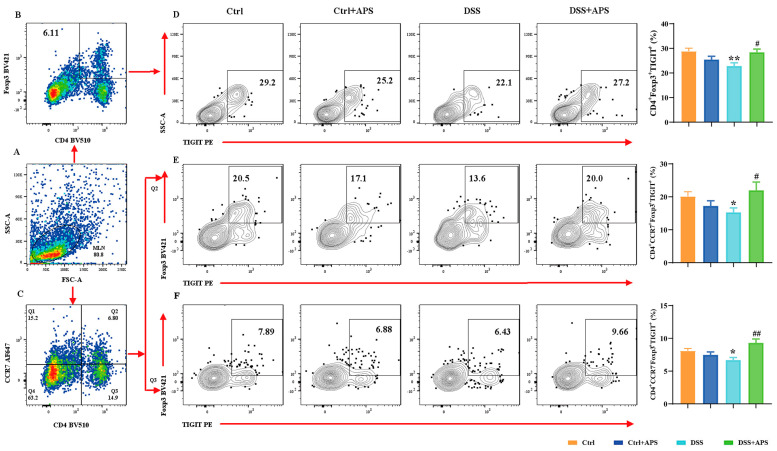
Flow cytometry analysis of the expression of TIGIT on the surface of memory Treg cells. (**A**) Total number of lymphocytes in representative samples. (**B**) CD4^+^Foxp3^+^ cells (Treg) in representative samples (**D**) CD4^+^Foxp3^+^TIGIT^+^ cells (TIGIT^+^mTreg) in representative samples. (**C**) CD4^+^CCR7^+^/CD4^+^CCR7^−^ cells in representative samples. (**E**) CD4^+^CCR7^+^Foxp3^+^TIGIT^+^ cells (TIGIT^+^cmTreg) in representative samples. (**F**) CD4^+^CCR7^−^Foxp3^+^TIGIT^+^ cells (TIGIT^+^emTreg) in representative samples. Data are expressed as mean ± SEM (*n* = 10). Significantly different compared to the Ctrl group (* *p* < 0.05, ** *p* < 0.01). Significantly different compared to the DSS group (^#^ *p* < 0.05, ^##^ *p* < 0.01).

TIGIT is an effective immune checkpoint protein expressed on effector T cells, Th cells and Treg cells [31]. Here, we found that CD4^+^CCR6^+^TIGIT^+^ (Figure 4D), CD4^+^CCR7^−^CCR6^+^TIGIT^+^ (Figure 4E) and CD4^+^CCR7^−^CCR6^+^TIGIT^+^ (Figure 4F) cells were significantly higher in the DSS group than in the Ctrl group, while CD4^+^Foxp3^+^TIGIT^+^ (Figure 5D), CD4^+^CCR7^+^Foxp3^+^TIGIT^+^ (Figure 5E) and CD4^+^CCR7^−^Foxp3^+^TIGIT^+^ (Figure 5F) cells were significantly lower. At the same time, CD4^+^CCR6^+^TIGIT^+^ (Figure 4D), CD4^+^CCR7^−^CCR6^+^TIGIT^+^ (Figure 4E) and CD4^+^CCR7^−^CCR6^+^TIGIT^+^ (Figure 4F) cells in MLNs were significantly higher in the DSS+APS group than in the DSS group, while CD4^+^Foxp3^+^TIGIT^+^ (Figure 5D), CD4^+^CCR7^+^Foxp3^+^TIGIT^+^ (Figure 5E) and CD4^+^CCR7^−^Foxp3^+^TIGIT^+^ (Figure 5F) cells were significantly lower. These results indicated that mTh17/mTreg cells aberrantly expressed TIGIT, and APS was effective in reversing TIGIT expression in mTh17/mTreg cells of the colitis mice.

### 2.4. APS Inhibits Activation of the TIGIT/CD155 Signaling Pathway in Mice with Colitis

**Figure 6:**
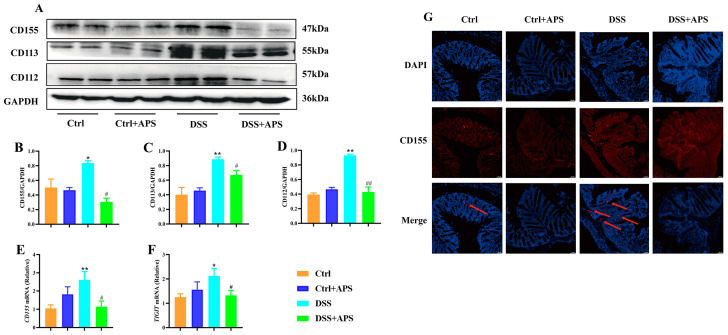
APS inhibits the activation of the TIGIT/CD155 signaling pathway in colitis mice. (**A**) Expression of CD155, CD113 and CD112 in colonic tissues was analyzed with Western blotting, and GAPDH was used as a reference for the whole protein. (**B**–**D**) Quantitative evaluation of CD155, CD113 and CD112 using Image J to determine spectral band strength. (**E**,**F**) Expression of CD155 and TIGIT in colonic tissue was analyzed with RT-PCR with GAPDH as an internal reference gene. (**G**) Immunofluorescence staining with CD155 (red) and DAPI (blue), red arrows indicate TIGIT-positive cells. Data are expressed as mean ± SEM (*n* = 4). Significantly different compared to the Ctrl group (* *p* < 0.05, ** *p* < 0.01). Significantly different compared to the DSS group (^#^ *p* < 0.05, ^##^ *p* < 0.01).

TIGIT is expressed in CD4^+^ T cells and Treg cells and suppresses adaptive immunity through TIGIT signaling, and CD155, CD113 and CD112 are the high-affinity homologous receptors for TIGIT [20]. Here, we found that the levels of CD155 (Figure 6A,B), CD113 (Figure 6A,C) and CD112 (Figure 6A,D) proteins were significantly higher in the colonic tissues of the colitis mice in the DSS group than in the Ctrl group, the mRNA levels of CD155 (Figure 6E) and TIGIT (Figure 6F) were also significantly higher, and a stronger CD155-positive signal in the tissue of the lamina propria was observed under fluorescence microscopy (Figure 6G). This indicated that the TIGIT/CD155 signaling was activated abnormally in the colonic tissues of the colitis mice. Importantly, the levels of CD155 (Figure 6A,B), CD113 (Figure 6A,C) and CD112 (Figure 6A,D) proteins were significantly lower in the colon tissues of the DSS+APS group than in the DSS group, the mRNA levels of CD155 (Figure 6E) were significantly lower while those of TIGIT (Figure 6F) were significantly higher, and CD155-positive cells were less distributed in the colonic lamina propria tissues (Figure 6G). These results suggested that APS effectively inhibited the activation of TIGIT/CD155 signaling in the colonic tissues of the mice with colitis.

### 2.5. APS Inhibits the Activation of PI3K/AKT Signaling Pathway in Mice with Colitis

**Figure 7:**
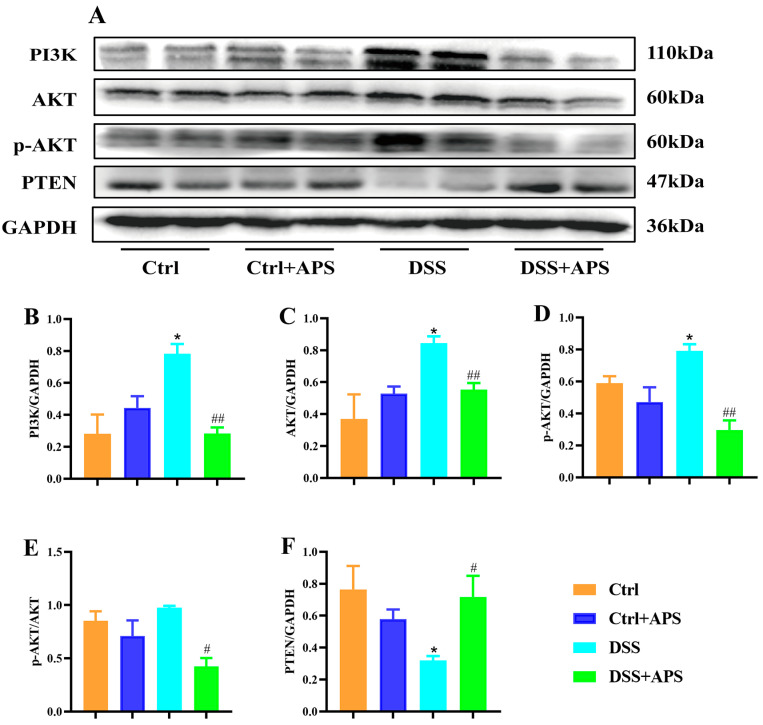
APS inhibits the activation of PI3K/AKT signaling pathways in colitis mice. (**A**) Expression of PI3K, AKT, p-AKT and PTEN in colonic tissue was analyzed with Western blotting, and GAPDH was used as a reference for the whole protein. (**B**–**F**) Quantitative evaluation of PI3K, AKT, p-AKT and PTEN using Image J to determine spectral band strength. Data are expressed as mean ± SEM (*n* = 3). Significantly different compared to the Ctrl group (* *p* < 0.05). Significantly different compared to the DSS group (^#^ *p* < 0.05, ^##^ *p* < 0.01).

TIGIT also inhibits the downstream PI3K/Akt signaling pathway directly through its intracellular ITIM structural domain, thereby delivering inhibitory signals to the T cell interior. Here, PI3K (Figure 7A,B), AKT (Figure 7A,C) and p-AKT (Figure 7A,D) protein levels in the colon tissues were significantly higher in the DSS group than in the Ctrl group, while PTEN (Figure 7A,F) protein levels were significantly lower. Critically, PI3K (Figure 7A,B), AKT (Figure 7A,C) and p-AKT (Figure 7A,D) protein levels and p-AKT/AKT (Figure 7A,E) were significantly lower in the colonic tissues of the DSS+APS group than in the DSS group, while PTEN (Figure 7A,F) protein levels were significantly increased. These results demonstrated that APS effectively inhibited PI3K/AKT signaling activation in the colonic tissues of the mice with colitis.

## 3. Discussion

Th17 cells secrete pro-inflammatory cytokines, such as IL-22, IL-17 and IFN-γ, to exacerbate the inflammatory response, while Treg cells secrete anti-inflammatory cytokines IL-35, IL-10 and TGF-β to exert anti-inflammatory effects [32,33]. As acute inflammation subsides, a small proportion of the effector survive and convert to memory Th17 cells (mTh17), which sustain chronic inflammation in autoimmune diseases [34]. CD patients presented higher frequencies of CCR6^+^CXCR3^-^RORγ^+^Tbet^-^CD4^+^ (Th17) memory T cells enriched in CD62L^low^ effector memory T cells (TEM) compared to healthy individuals [35]. Basophils increase in Crohn’s disease and UC and favor the mTh17/mTh1 response in MLNs [12]. There was a significant increase in the frequencies of CD4^+^CCR7^−^Foxp3^+^ and CD4^+^CCR7^−^IL-10^+^ mTreg cells and a significant decrease in CD4^+^CCR7^−^IL-17A^+^ mTh17 cells in the MLNs of the mice with DSS-induced experimental colitis [36]. In the present study, we also found that the colitis mice presented similar results with lower frequencies of mTreg (CD4^+^Foxp3^+^, CD4^+^CCR7^+^Foxp3^+^, CD4^+^CCR7^−^Foxp3^+^) cells and higher mTh17 (CD4^+^CCR6^+^, CD4^+^CCR7^+^CCR6^+^, CD4^+^CCR7^−^CCR6^+^) cells.

APS is the main bioactive component extracted from *Astragalus membranaceus*, which has various pharmacological effects, such as immune modulation, cardiovascular protection, anti-tumor, metabolic improvement and anti-inflammation, and is widely used in the treatment of autoimmune diseases, cancer and other diseases [37,38]. There are studies demonstrating that APS is effective in alleviating UC. In a pivotal study, Tian Z et al. discovered that dosages of APS at 100, 200 and 500 mg/kg can impede the activation of the NLRP3 inflammasome, which leads to reduced production of IL-18 and IL-1β, culminating in therapeutic outcomes for DSS-induced colitis [39]. Meng et al. validated that varying concentrations of APS (50, 100 and 200 μg/mL) effectively halted the production of pro-inflammatory cytokines by inhibiting the PI3K/AKT/mTOR pathway in vitro [40]. Previously, our research group discovered that APS (400 mg/kg) significantly increased the proportion of Treg cells in the Peyer’s patches of rats induced with TNBS colitis [28]. Additionally, Zhong et al. reported that 200 mg/kg APS can modulate the expression of inflammatory cytokines, such as IL-2, IL-6, IL-12p70, IL-23 and TNF-α, in the colonic tissues of mice with colitis, rebalance the homeostasis between Tfh/Treg cells [29] and recalibrate the balance of Tfh/Treg cells, but it had no significant effect on Th17 cells. Differing from the aforementioned findings, our research showed that 10 days of APS treatment could effectively modulate the balance of Th17/Treg cells in the colitis mice. The variance in effects regarding the regulation of Th17 cells by APS may be due to the different mouse strains used and the duration of the APS administration—7 days in C57BL/6 mice versus 10 days in BALB/c mice. More crucially, we have demonstrated, for the first time, that APS can effectively treat UC by influencing the balance between memory Th17/memory Treg cells in close relation with the modulation of the TIGIT/CD155 signaling pathway.

Human Th17 cells represent a distinct subset of long-lived, proliferative effector memory T cells with unique genetic and functional characteristics [41]. Increased frequencies of these memory Th17 cells, which express CCR6, CXCR3, RORγ and T-bet and contain CD62L effector memory T cells, have been observed in the MLNs of patients with UC [42]. Studies have shown that Treg cells, isolated from human peripheral blood and cultured in vitro for 6 weeks, exhibit pronounced memory phenotypes and immunosuppressive capacities [43]. Vedolizumab, a novel biologic agent primarily targeting patients with IBD, especially for the clinical treatment of UC, has been researched in clinical trials. Observations from these studies include significant transcriptional differences in CD4^+^ memory T cells and Tregs in the ileum and colon of IBD patients treated with Vedolizumab with particularly increased expression of genes related to Treg oxidative phosphorylation, which may enhance Treg cell function [44]. Another study found that the clinical response in IBD patients treated with Vedolizumab for 14 weeks is closely correlated with the baseline levels of inherent mucosal memory Th17 and Th1/17 cells prior to treatment with the patients exhibiting lower baseline levels tending to have better endoscopic responses. [35] In this context, APS regulation of the mTh17/mTreg cell balance in mice with DSS-induced colitis has been proven effective, demonstrating the potential effects of APS in treating UC. However, further clinical research is necessary to confirm its therapeutic value.

The TIGIT/CD155 signaling pathway is closely associated with the development and progression of UC [6]. In IBD patients with active intestinal inflammation, the activation of CD25^+^CD45A^+^ Treg cells by circulating CD38^+^effector T cells increased, and the frequency of TIGIT^+^ cells decreased [45]. Importantly, TIGIT plays a role in modulating the function of Foxp3^+^ Treg cells, which is significant for achieving clinical remission in UC patients [46]. In addition, UC patients have a reduced number of peripheral NK cells and an altered phenotype of these cells that includes increased TIGIT expression [47]. In the present study, a significant decrease in TIGIT levels on mTreg cells and a significant increase in TIGIT levels on mTh17 cells were found in the colitis mice. This suggests that significant abnormal changes in TIGIT occurred when the balance between mTh17 and mTreg cells was disrupted, which was closely related to their balance. Meanwhile, we found that APS could effectively enhance the expression of TIGIT on mTreg cells while inhibiting the expression on mTh17 cells. In addition, the TIGIT downstream signaling PI3K/AKT pathway is an important intracellular signaling pathway [7,48]. Notably, TIGIT can block the activation of the PI3K and AKT pathways, thereby inhibiting cytokine secretion and improving T cell survival [49]. Targeted regulation of PI3K/Akt signaling to reshape the balance of Th17/Treg cells is an effective measure for the treatment of autoimmune diseases [50,51]. Extracellular vesicles produced by bone marrow mesenchymal stem cells overexpressing PD-L1 ameliorated DSS-induced UC in rats by regulating the Th17/Treg cell balance through the PTEN/PI3K/AKT/mTOR axis [52]. Similarly, Compound 511 mitigates MRSA-induced lung injury in mice by countering morphine-induced immunosuppression through the PI3K/AKT/mTOR pathway [53]. Importantly, we found that APS not only regulated TIGIT expression in mTh17/mTreg cells but also inhibited TIGIT/CD155 signaling activation in colonic tissues.

Our study has provided insight into the therapeutic potential of APS for the treatment of colitis. We have demonstrated that APS can modulate the homeostasis of mTh17/mTreg cells, which is intricately linked to TIGIT/CD155 signaling. Despite these findings, our research has certain limitations that warrant further exploration. However, whether APS relies on TIGIT/CD155 signaling to regulate mTh17/mTreg cell differentiation, effector functions need further investigation. However, the mTh17/mTreg cell-specific marker IL-17A/Foxp3 is an intracellular molecule, resulting in the inability to directly access large numbers of mTh17/mTreg cells. Meanwhile, some studies have reported that CD73 and CD39 are membrane-specific markers for mTh17 and mTreg cells, respectively, and we are conducting related studies and may see our new studies reported in the near future. If mTh17/mTreg cells can be directly obtained in vitro, siRNA technology silences or overexpresses TIGIT/CD155 signaling to further probe the APS-dependent TIGIT/CD155 pathway regulating mTh17/mTreg cell homeostasis.

## 4. Materials and Methods

### 4.1. Animals

Male BALB/c mice (SPF, 6–8 weeks, 22 ± 2 g) were purchased from Hunan Slake Jingda Experimental Animal Co., Ltd. (Changsha, China) (license number: SCXK (Xiang) 2019-0004). The mice were housed in a class 10,000 barrier environment (23 ± 2 °C, 45–65% humidity) at the Animal Center of Jiangxi University of Chinese Medicine. The experimental protocols were approved by the Animal Care and Use Committee of Jiangxi University of Chinese Medicine (Approval number: JZLLSC20210280; Date: 21 June 2021).

### 4.2. Drugs and Reagents

APS (Formula: C_10_H_7_ClN_2_O_2_S; No. 89250–26-0; purity > 98%) was purchased from Macklin (Shanghai, China). DSS (#160110, MW: 36–50 kDa) was purchased from MP Biomedicals (Irving, TX, USA). The anti-mouse BV510-CD4 (#563106) and anti-mouse Alexa Fluor 647-CCR7 (#560766) for flow cytometry were purchased from BD Biosciences (Franklin Lakes, NJ, USA); anti-mouse BV421-Foxp3 (#48-5773-82) was purchased from ThermoFisher (Waltham, MA, USA) for flow cytometry analysis. The anti-rabbit GAPDH (#ab181602) and anti-rabbit PTEN (#ab170941) antibodies were purchased from Abcam (Waltham, MA, USA); anti-rabbit PI3K (#27921-1-AP) was purchased from Proteintech (Wuhan, China); anti-mouse AKT (#4685S) and anti-mouse p-AKT (#4060) were purchased from Cell Signaling Technology (Boston, MA, USA); anti-rabbit CD155 (#bs-2525R), anti-rabbit CD113 (#bs-6181R) and anti-rabbit CD113 (#bs-6181R) were purchased from BIOSS Company (Beijing, China); anti-mouse CD112 (#DF7313) was purchased from Affinity Biosciences (Changzhou, China) for Western blotting.

### 4.3. UC Model and APS Treatment

The UC mice model was replicated according to the method of reference [30]. After 3 days of acclimatization, the mice were randomly divided into 4 groups: Ctrl, Ctrl+APS, DSS and DSS+APS. The mice in the DSS and DSS+APS groups drank 3% DSS for 7 consecutive days to induce experimental colitis, while the mice in the Ctrl and Ctrl+DSS groups drank water normally. Simultaneously, the mice in the Ctrl+APS and DSS+APS groups were gavaged with 200 mg/kg/day of APS (pre-dissolved in saline) for 10 consecutive days [54], while the mice in the Ctrl and DSS groups were gavaged with equal volumes of saline.

### 4.4. Sample Collection and Colonic Index

Throughout the experiment, the mice were weighed and observed for clinical signs (fecal consistency, blood in the stool, etc.) at the same time each day. On day 11, the mice were euthanized under deep anesthesia with 2% sodium pentobarbital, the abdominal cavity was rapidly opened, fresh colon and MLNs tissues were collected, and the colon length was measured and weighed. The colon 2 cm from the ileocecal region was placed in 4% paraformaldehyde solution, and the remaining colon tissues were snap-frozen in liquid nitrogen and stored in a freezer at −80 °C. The colonic weight index and colonic weight/colonic length were calculated (Colonic weight index (%) = colonic weight (g)/mouse weight (g) × 100%).

### 4.5. Histopathological Analysis

Colon tissues were fixed in 4% paraformaldehyde for 7 days, graded alcohol dehydrated and xylene transparent, paraffin embedded and made into 4 μm thick sections. These sections were routinely stained with hematoxylin–eosin (H&E) and sealed with neutral gum, and the morphological changes in the colonic tissues were observed under a light microscope (Leica, BX43, Wetzlar, Germany) and pictures collected. Histopathological injury of the colon was scored randomly and blindly based on inflammation, extent of lesions, crypt destruction and lesion extent [55].

### 4.6. Flow Cytometry Analysis

Fresh mouse MLN tissues were taken in RPMI 1640 medium, ground, filtered and centrifuged, and stain buffer was re-suspended to prepare a single-cell suspension. The staining buffer-resuspended cells were centrifuged at 350 g/min for 5 min and washed twice. The FC block was incubated at room temperature for 15 min to block the non-specific sites. The flow cytometry surface-stained antibodies (CCR6, CCR7, CD4, TIGIT) were incubated for 30 min in the dark for cell membrane-specific staining. The cells were fixed and broken with the Cytofix/Cytoperm kits (BD Biosciences, Franklin Lakes, NJ, USA) and then incubated for 30 min in the dark with intracellular and nuclear antibodies (Foxp3) for intracellular labeling. Finally, FACSCanto II flow cytometry (BD Biosciences, Franklin Lakes, NJ, USA) was used to detect these stained cells. Negative controls were set up simultaneously to clarify cell fractionation and to exclude cell debris with gating. FlowJo 10.0 software was used to analyze these acquired data.

### 4.7. Western Blotting

Colon tissue (100 mg) was placed in RIPA buffer (Solarbio, Beijing, China) at a ratio of 1:10, and total protein from colon tissue was extracted with thorough grinding in a tissue homogenizer and incubated on ice for 30 min. The total protein concentration of each sample was determined with a BCA kit (Beyotime, Nanjing, China). Protein samples were mixed with loading buffer and boiled for 10 min, separated with electrophoresis on SDS-PAGE gels, electrotransferred to polyvinylidene fluoride (PVDF) membranes using a Bio-Rad protein blotter blocked with 5% BSA for 2 h at room temperature and then incubated with primary antibodies at 4 °C overnight. Primary antibodies included PI3K (1:1000), AKT (1:1000), p-AKT (1:2000), PTEN (1:10,000), CD155 (1:1000), CD113 (1:1000) and CD112 (1:1000). Then, the corresponding secondary antibodies, goat anti-rabbit lgG (HRP) (1:10,000) and goat anti-mouse lgG (horseradish peroxidase) (1:50,000), were incubated for 1–2 h at room temperature. Subsequently, protein bands were detected under a UVP Chen Studio (Analytik Jena, Upland, CA, USA) using an ECL protein blotting substrate (Solarbio, Beijing, China). The grayscale values of the protein bands were detected using Image Pro Plus 6.0 software (La Jolla, CA, USA), and the relative expression of the target protein (CD155, CD113, CD112, PI3K, AKT, p-AKT, PTEN) was calculated using GAPDH as an internal reference.

### 4.8. RNA Extraction and Real-Time PCR

Total RNA was extracted from mouse colon tissues according to the instructions of the M5 Universal RNA Mini Kit Release/Cellular RNA Rapid Extraction Kit (Jumei Biotechnology, Beijing, China). The quality of total RNA was detected with nucleic acid electrophoresis, and samples of better quality were selected for reverse transcription to cDNA according to the manufacturer’s instructions for the M5 Sprint qPCR RT kit with gDNA remover (Mei5bio, Beijing, China). The cDNA samples were used for real-time PCR with SYBR Green reagents on a bioanalyzer (Roche LightCyler96, Basel, Switzerland). The Ct values of each sample were performed in triplicate. The relative mRNA expression levels of TIGIT and CD155 were calculated using the 2^−ΔΔCt^ method with GAPDH as the housekeeping gene. The primers used in RT-PCR are shown in Table 1.

### 4.9. Immunofluorescence

Paraffin sections (4 μm) were dewaxed and rehydrated, endogenous peroxidase was eliminated with 3% H_2_O_2_, citrate buffer (pH = 6.0) was microwave boiled for 10 min for antigen repair, BSA was used to block non-specific protein binding sites, and the primary antibody CD155 (1:100) was incubated overnight at 4 °C in a dark room. The next day, the corresponding fluorescent secondary antibody was anti-mouse lgG (1: 400). Subsequently, secondary antibodies anti-mouse lgG (1: 400) were used to fluorescently label the TIGIT complexes of the sectioned tissues. DAPI re-stained sections were glycerol sealed. Ultimately, the sections were observed under a fluorescent microscope (Leica, DM2000LED, Wetzlar, Germany), and corresponding pictures were acquired.

### 4.10. Statistical Analysis

GraphPad Prism 8.0 software (La Jolla, CA, USA) was used for the statistical analysis. All data were analyzed with two-way analysis of variance (ANOVA) followed by Fisher’s LSD post hoc tests. The results were expressed as mean ± standard error of the mean (SEM), and the differences were statistically significant at *p* < 0.05.

## 5. Conclusions

**Figure 8:**
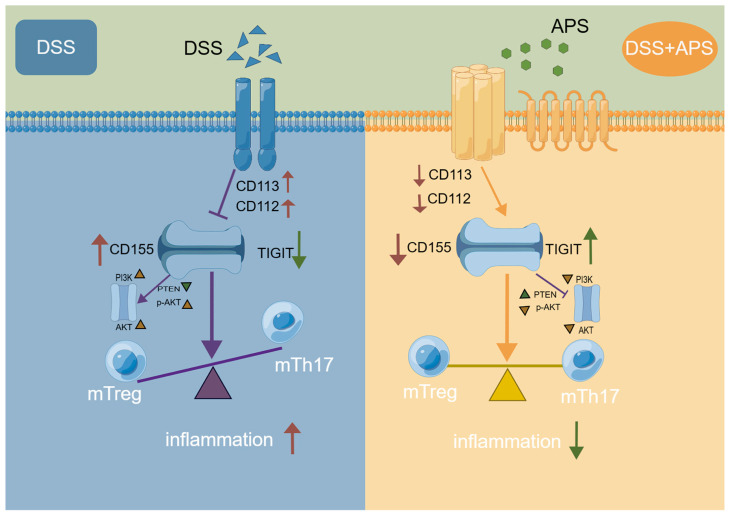
Potential action mechanism of APS against colitis in mice.

We demonstrated for the first time that DSS-induced colitis mice presented the imbalance of mTh17/mTreg cells with higher levels of mTh17, cmTh17 and emTh17 cells and lower levels of mTreg, cmTreg and emTreg cells in the MLNs. Importantly, APS effectively alleviated UC by reshaping the balance of mTh17/mTreg cells in association with regulation of TIGIT/CD155 signaling (Figure 8). The data strongly suggest APS’s therapeutic promise for UC treatment, highlighting the need for urgent further investigations. Such studies should focus on clarifying the molecular mechanisms behind APS’s modulation of mTh17/mTreg cells and TIGIT/CD155 signaling, evaluating the long-term efficacy and safety of APS in UC and determining the translational potential of these findings to human UC pathology with the ultimate goal of developing novel APS-based therapies for UC management.

## Figures and Tables

**Table 1:** Primers used for RT-PCR.

Primers	Sequence	
GAPDH	Forward	5′-TGGTGAAGGTCGGTGTGAAC-3′
	Reverse	5′-TGAATTTGCCGTGAGTGGAG-3′
TIGIT	Forward	5′-CTGATACAGGCTGCCTTCCT-3′
	Reverse	5′-TGGGTCACTTCAGCTGTGTC-3′
CD155	Forward	5′-CCAGTGAGCACTCAGGTACA-3′
	Reverse	5′-GTCTGTGGATCCTGGGAAGA-3′

## Data Availability

The data underlying this article will be shared on reasonable request to the corresponding author.
